# Antimicrobial efficacy of 0.8% Hyaluronic Acid and 0.2% Chlorhexidine against *Porphyromonas gingivalis* strains: An in-vitro study

**DOI:** 10.12669/pjms.36.2.1456

**Published:** 2020

**Authors:** Munerah Binshabaib, Kawther Aabed, Fitoon Alotaibi, Milaf Alwaqid, Aljohara Alfraidy, Shatha Alharthi

**Affiliations:** 1 Munerah Binshabaib, BDS, MSc. Department of Preventive Dental Sciences, College of Dentistry, Princess Nourah Bint Abdulrahman University, Riyadh, Saudi Arabia; 2 Kawther Aabed, BDS. Department of Biology, Faculty of Sciences, Princess Nourah Bint Abdulrahman University, Riyadh, Saudi Arabia; 3 Fitoon Alotaibi, BDS. General Dental Practitioner, Private Dental Practitioners, Riyadh, Saudi Arabia; 4 Milaf Alwaqid, BDS. General Dental Practitioner, Private Dental Practitioners, Riyadh, Saudi Arabia; 5 Aljowhara Faraidy, BDS. General Dental Practitioner, Private Dental Practitioners, Riyadh, Saudi Arabia; 6 Shatha Alharthi, BDS, MSc. Department of Preventive Dental Sciences, College of Dentistry, Princess Nourah Bint Abdulrahman University, Riyadh, Saudi Arabia

**Keywords:** Bacteria, Chlorhexidine gluconate, Hyaluronic acid, *In-vitro*, *Porphyromonas gingivalis*

## Abstract

**Objective:**

The aim of the present in-vitro study was to assess antimicrobial efficacy of 0.8% hyaluronic acid (HA) and 0.2% Chlorhexidine gluconate (CHX) against Porphyromonas gingivalis (P. gingivalis).

**Methods:**

The study was performed between December 2018 and March 2019 at the College of Dentistry at the Princess Nourah Bint Abdulrahman University, Riyadh, Saudi Arabia. The P. gingivalis biofilms were formed and grown for 72 hours at 37°C under anaerobic conditions on glass slides coated with human saliva. The slides were individually positioned and exposed to 0.8% HA or 0.2% CHX. Therapeutically, the biofilms were divided into 3 groups as follows: (a) negative group; (b) 0.8% HA group and (c) 0.2% CHX group. P-values less than 0.05 were considered statistically significant.

**Results:**

In the 0.8% HA group, P. gingivalis CFUs/ml were significantly higher at baseline than at 24- (P<0.05), 48 (P<0.05) and 72 hours (P<0.05) intervals. In the 0.2% CHX group, P. gingivalis CFUs/ml were significantly higher at baseline than at 72 hours interval (P<0.05). In the CHX group, there was no difference in P. gingivalis CFUs/ml between baseline, 24- and 48-hours intervals. At 48- and 72-hours intervals, the P. gingivalis CFUs/ml were significantly higher in the 0.2% CHX group compared with the 0.8% HA group.

**Conclusion:**

In-vitro, 0.8% HA is more effective in reducing the P. gingivalis CFUs/ml compared with 0.2% CHX.

## INTRODUCTION

Periodontitis is associated with inflammatory conditions including gingival inflammation, increased probing depth, clinical attachment loss; and resorption of supporting alveolar bone.^1^ From a clinical perspective, poor oral hygiene maintenance is a well-known and significant risk-factor of periodontitis;^2^ however, laboratory-based investigations on oral biofilm samples collected from patients with periodontitis have shown an increased colonization of pathogenic microbes including *Porphyromonas gingivalis* (*P. gingivalis*), *Treponema denticola* (*T. Denticola*), and *Tannerella forsythia* (*T. forsythia*), collectively known as Red Complex Bacteria (RCB).^3^ The RCB are well-known microbes associated with the etiopathogenesis of periodontal inflammatory conditions, including periodontitis and triggers inflammatory signaling pathways thereby jeopardizing human gingival fibroblasts.^4^

Hyaluronic acid (HA) is a high molecular weight (20,000 kilodaltons) polysaccharide that belongs to the family of glycosaminoglycans.^5^ It consists of glucuronic acid, N-acetyl-glucosamine and a basic unit of two sugars. HA commonly exists in the synovial fluid, cartilage, and tissues of the eye and skin.^5^ The high molecular weight of HA exerts immunosuppressive and anti-inflammatory effects and promotes wound healing.^5^ It has also been reported that HA is present in the extracellular matrix of periodontal tissues and plays a role in maintaining a healthy periodontium.^6^ In a clinical study, Vanden Bogaerde L.^7^ investigated the efficacy of esterified HA in the treatment of periodontitis. The results showed that application of HA is a reliable therapeutic regimen for the treatment of infrabony periodontal defects.^7^ Moreover, results of a recent *in-vitro* study^8^ showed that HA inhibits *P. gingivalis*-induced interleukin (IL)-1β, IL-4, IL-6, IL-8, and IL-10 production in a dose-dependent manner. Chen et al.^8^, proposed that HA has benefits periodontal tissues by reducing inflammation and its related parameters and promoting mound healing.

It has been shown that CHX application has antimicrobial effect against various bacteria including *Enterococcus Faecalis*, *Prevotella Intermedia*, *P. gingivalis* and *Staphylococcus aureus*.^9^ However, there are no studies that have compared the antimicrobial efficacy of HA with 0.2% CHX against the *P. gingivalis*. It is hypothesized that HA and CHX exhibit a comparable level of antimicrobial efficacy against RCB. The aim of the present *in-vitro* experiment was to compare the antimicrobial efficacy of 0.8% HA with 0.2% CHX against *P. gingivalis*.

## METHODS

### Ethical statement

The study protocol was reviewed and approved by the Research Ethics Review committee IRB No. 18-0320 dated November 29, 2018 of the Princess Nourah Bint Abdulrahman University, Riyadh, Saudi Arabia. The study was performed between December 2018 and March 2019 at the College of Dentistry, Princess Nourah Bint Abdulrahman University, Riyadh, Saudi Arabia. Since the present study had an experimental design, a consent form was not warranted.

### Bacterial strain

The *P. gingivalis* strains (*ATCC*^®^ *33277*^™^ Manassas, VA,USA) were grown and maintained as described elsewhere.^10^ In summary, *P. gingivalis* W83 biofilms were formed and grown anaerobically on glass-slides, which were coated with human saliva for 96 hours at 37 °C. Each slide was positioned in 50 mL tubes containing 45 mL of the bacterial inoculum (1.5 × 108 bacteria/mL) with Brain-Heart-Infusion broth supplemented with 5 µg/mL hemin and 1 µg/mL menadione. The slides were randomly exposed to either 0.8% HA or 0.2% CHX. The culture medium was replaced daily.

### Treatments, grouping and bacterial viability

A vertical thin strip containing 0.8% HA (Group-1), 0.2% CHX (Group-2) was individually positioned in 50 mL tubes containing 45 mL of fresh culture medium for *P. gingivalis*. The negative-control (Group-3) comprised of a biofilm group which was not exposed to any formulation. The biofilm and culture medium were collected at 24, 48, and 72 h after exposure to the 0.2% CHX and 0.8% HA formulations. The pH of the culture medium was also measured using a digital device (GEHAKA, pHmetro de bancada PG2000, São Paulo, Brazil) prior to CHX or HA quantification. After 24, 48 and 72 hours, biofilms were collected using a sterile swab. Each biofilm was sonicated after being transferred into a microcentrifuge tube that contained 0.9% NaCl (1 ml).^11^ Each biofilm suspension was serially diluted and inoculated in BHI-agar blood plates (supplemented with 5 g/mL hemin and 1 g/mL menadione) for the growth of *P. gingivalis*.^11^ Colony-forming units (CFUs) were measured and recorded in CFUs/mm^2^.

### Statistical analysis

Statistical analysis was performed using a software program (SPSS version 20, Chicago, IL., USA). Assumptions of equality of variances and normality distribution of errors was checked. Bacterial viability of *P. gingivalis* was statistically assessed using the Tukey test. P-values below were considered statistically significant.

## RESULTS

At baseline, the CFUs/ml were comparable in the study groups. There was no statistically significant difference in the *P. gingivalis* CFUs/ml in the negative control group at all-time intervals. In the 0.8% HA group, *P. gingivalis* CFUs/ml were significantly higher at baseline compared with microbial colonization at 24- (P<0.05), 48- (P<0.05) and 72 hours (P<0.05) intervals. In the 0.2% CHX group, *P. gingivalis* CFUs/ml were significantly higher at baseline compared with microbial CFUs/ml at 72 hours interval (P<0.05). In the CHX group, there was no statistically significant difference in the *P. gingivalis* CFUs/ml at baseline and at 24- and 48 hours’ intervals (Table-I). At 48- and 72 hours’ intervals, the *P. gingivalis* CFUs/ml were significantly higher in the 0.2% CHX group compared with the 0.8% HA group (P<0.05) (Table-I).

**Table-I:** Colony forming units (CFUs/ml) of *Porphyromonas gingivalis* at baseline and at 24-, 24- and 72 hours’ intervals in the study groups.

Groups	Colony forming units per milliliter (1x10^9^)			

	Baseline	24 hours	48 hours	72 hours
Negative control	1.6 ± 0.3	1.72 ± 0.5	1.81 ± 0.4	1.92 ± 0.5
0.8% HA Group	1.6 ± 0.4	1.1 ± 0.08	0.6 ± 0.02*	0.4 ± 0.01*
0.2% CHX group	1.6 ± 0.3	1.4 ± 0.4	1.2 ± 0.07	1 ± 0.05

* Compared with 0.2% CHX (P<0.05).

## DISCUSSION

It is well-established that CHX exhibits antibacterial properties and is a useful adjunct to traditional treatments of CP, such as scaling and root planning (SRP).^12^ However, the present experimental results indicate that the potency of 0.8% HA to reduce the counts of periodontopathogenic microbes is higher than that of CHX. One explanation for this is that hydrophilicity of HA enhances the receptiveness of coagulum thereby improving cell repair, differentiation and proliferation of basal keratinocytes and mesenchymal cells.^13^ Moreover, HA facilitates osseous regeneration via induction of osteogenic proteins such as osteopontin and bone morphogenetic protein-2.^14^ Furthermore, the high concentration of medium and lower molecular weight HA exhibits bacteriostatic properties against a variety of pathogenic microbes including *Prevotella Oris*, *Aggregatibacter actinomycetemcomitans* and *Streptococcus* species;^14^,^15^ and the present *in-vitro* experiment is among the limited evidence that has shown HA to exbibit antibacterial effect against *P. gingivalis*.

From a clinical perspective, it is speculated that use of HA-based oral rinses when used as an adjunct to mechanical debridement (synonym, SRP) is more effective in the treatment of CP as compared to when CHX-based mouthwashes are used with SRP. The authors of the present experimental study support the results of a split-mouth randomized clinical trial (RCT)^16^ in which, 24 patients with moderate to severe CP were evaluated after SRP. In this study, the test-sites received 0.8% HA gel application as an adjunct to SRP and the control-sites underwent SRP alone. The 12-week follow-up results showed that there was a statistically significant reduction in periodontal inflammation in the test- compared with the control-sites.^16^ The authors of the present study hypothesize that the 0.8% HA gel significantly reduced the counts of pathogenic microbes (including *P. gingivalis*) in the test- compared with the control-sites thereby markedly reducing the clinical markers of periodontal inflammation (including plaque index, gingival bleeding and pocket depth). The authors also speculate that SRP when used as an adjunct to SRP is more effective in the treatment of CP in medically compromised patients such as individuals with chronic hyperglycemia. It is well-established that persistent hyperglycemia (such as among patients with poorly controlled diabetes mellitus and prediabetes) is a risk factor of periodontal and peri-implant diseases^17^,^18^; and a state of chronic hyperglycemia delays wound healing after periodontal therapy.^19^,^20^ According to Bansal et al.^14^, hyaluronate modulates wound healing and its administration in sites with periodontal defects helps. Moreover, in a recent study in diabetic rats, Eliezer et al.^21^ showed that cross-linked HA augments osseous wound healing by slowing down collagen membrane degradation. Further well-designed randomized controlled clinical trials are needed to test these aforementioned experimental results.^14^,^21^

### Limitations of the study

One limitation of the present study is that the results were entirely based on an *in-vitro* assessment of *P. gingivalis* strains. This makes it difficult to contemplate these laboratory-based results into a clinical setting in which, confounders such as habitual tobacco smoking and an immunocompromised health status may potentially compromise the efficacy of SRP with or without adjunct HA therapy in patients with CP. Moreover, only one concentration of HA (0.8%) was tested. The minimum concentration of HA that may potentially help reduce periodontal inflammation and augment healing remains to be determined. This warrants additional studies.

## CONCLUSION

The 0.8% HA is more effective in reducing the *P. gingivalis* CFUs/ml compared with 0.2% CHX. Further well-designed RCTs are needed to assess the clinical efficacy of 0.8% HA as an adjunct to SRP in the treatment of CP.

### Authors’ Contribution:

**MB and SA** conceived and designed the study and edited the manuscript; and are responsible and accountable for the accuracy or integrity of the work.

**KA** wrote the methods and did statistical analysis.

**FA, MA & AA** did data collection and manuscript writing.

**SA and MB** did review and final approval of manuscript.
